# Editorial: *Actinobacteria*, a Source of Biocatalytic Tools

**DOI:** 10.3389/fmicb.2019.00800

**Published:** 2019-04-16

**Authors:** Dirk Tischler, Willem J. H. van Berkel, Marco W. Fraaije

**Affiliations:** ^1^Microbial Biotechnology, Biology and Biotechnology, Ruhr University Bochum, Bochum, Germany; ^2^Laboratory of Biochemistry, Wageningen University & Research, Wageningen, Netherlands; ^3^Molecular Enzymology, University of Groningen, Groningen, Netherlands

**Keywords:** actinomycetes, secondary metabolites, high GC genetics, novel biocatalysts, extremophile actinobacteria, biotechnology, biocatalysis, germination

## Actinobacteria: Ancient Phylum With Large Biotechnological Potential Still to be Uncovered

Actinobacteria (Actinomycetes) represent one of the largest and most diverse phyla among the Bacteria. The characteristics and phylogeny of actinobacteria have been well-described throughout the years (Anteneh and Franco; Embley et al., 1994; Stackebrandt et al., 1997a,b; Stach and Bull, 2005; Stackebrandt and Schumann, 2006; Ventura et al., 2007; Gao and Gupta, 2012; Goodfellow, 2012a,b; Schrempf, 2013; Lawson, 2018; Lewin et al., 2016). Still actinobacteria are hotspots for discovery of new biomolecules and enzyme activities, fueling an active field of research. The remarkable diversity is displayed by various lifestyles, distinct morphologies, a wide spectrum of physiological and metabolic activities, as well as genetics.

Most actinobacteria have a high GC-content (ranging from 51% to over 70%) and belong to Gram-positive or Gram-variable type microbes (Stackebrandt and Schumann, 2006; Ventura et al., 2007; Lawson, 2018). Many species are well-known for their large genomes, which may be of linear style, as in case of rhodococci, or circular (Ventura et al., 2007; Sen et al., 2014; Lewin et al., 2016). Many also harbor linear megaplasmids as a kind of genetic storage device (König et al., 2004; Medema et al., 2010; Wagenknecht et al., 2010; Bottacini et al., 2015). These plasmids often encode special metabolic features such as secondary metabolite synthetic machineries or alternative degradation pathways. However, a number of representatives comprise smaller genomes such as some *Bifidobacteria, Corynebacteria, Mycobacteria*, and *Propionibacteria* species (Ventura et al., 2007; Lewin et al., 2016). Interestingly, smaller genomes are often encountered in pathogens or in those, which live in ecological niches. The smallest actinobacterial genomes can be found among *Tropheryma*, which is known as the Whipple's disease microbe (Bentley et al., 2003; Raoult et al., 2003). Gene redundancy or genes encoding for closely related enzymes are frequently reported and in most cases the evolutionary history or a functional role remains enigmatic (McLeod et al., 2006; Tischler et al., 2009, 2010, 2013; Roberts et al., 2011; Riebel et al., 2012; Gröning et al., 2014; Riedel et al., 2015a,b; Nguyen et al., 2017; Chen et al., 2018; Gran-Scheuch et al.). In this context horizontal gene transfer was found to play a major role in the genome fluidity of actinobacteria (Ventura et al., 2007). However, this seems not to be true for all actinobacteria or limited to some features such as secondary metabolism as discussed for *Streptomyces* and *Rhodococcus*, respectively (McLeod et al., 2006; Lewin et al., 2016).

The large actinobacterial genomes and megaplasmids provide access to an impressive number of potential biocatalysts and pathways (Lewin et al., 2016). A few examples of novel biocatalysts linked to gene redundancy are cited above, but still more truly novel enzymes or pathways await elucidation. Actinobacteria are well-known for their biotechnological potential which is exemplarily described for amino acid producing *Corynebacteria* (Poetsch et al., 2011; Goldbeck et al.; Pérez-García et al.), secondary metabolite producing *Streptomyces* (Niu et al., 2016; Senges et al., 2018), pathogenic targets as *Nocardia* and *Mycobacteria* (Cosma et al., 2003; Wilson, 2012), carotenoid building *Micrococcus* strains (Rostami et al., 2016), acid fermenting *Propionibacteria* (Rabah et al., 2017), health and food related *Bifidobacterium* strains (Lawson, 2018), rubber degrading *Gordonia* species (Linos et al., 1999; Heine et al., 2018), and organic pollutant degrading rhodococci (McLeod et al., 2006; Kim et al., 2018) among others.

In many cases individual pathways can be exploited for the production of valuable products, or enzymes can be recombinantly produced and exploited for biocatalysis. Even some genetic tools to work directly in actinobacteria have been successfully used as for example in *Corynebacterium* (Nešvera and Pátek, 2011). Recently some additional systems have been established to create e.g., *Kocuria* and *Rhodococcus* hosts (Montersino et al.; Toda and Itoh). The first system allowed actually to express genes of various origins in *Kocuria*, whereas the *Rhodococcus* system was used for identification of the natural phospholipid ligand of a monooxygenase. During the last decade more and more genomes have been sequenced and made available for data mining and become accessible by state-of-the-art genomic manipulation methods. Novel pathways and enzymes are frequently described from actinobacteria as a result of the progress in various omics approaches and high-throughput methods. Except for novel pathways or enzymes, genome analyses have revealed that actinobacteria also employ rather unique cofactors, such as the F_420_ cofactor (Selengut and Haft, 2010; Greening et al., 2016; Nguyen et al., 2017; Ney et al.). With respect to biocatalysis and derived applications a number of recent studies can be mentioned. These comprise whole-cell systems (Oelschlägel et al., 2015; Okamoto et al., 2017; de Carvalho, 2017; Goldbeck et al.; Yin et al.) enzymatic cascades (Kara et al., 2015; Ni et al., 2016; Zimmerling et al., 2017), structure-function relationships (Riebel et al., 2012; Montersino et al., 2013; Riedel et al., 2015a,b; Sucharitakul et al., 2016; Scholtissek et al., 2017; Scholtissek et al.) as well as mechanistic insights (Greening et al.; Ney et al.; Westphal et al.).

Secondary metabolite production is of industrial interest and here especially *Streptomyces* has to be mentioned which provides access to antibiotics as well as siderophores (Medema et al., 2010; Čihák et al.; Botas et al.; López-García et al.; Senges et al., 2018; Suárez Moreno et al.). Secondary metabolite production is frequently investigated either on a regulatory level (Botas et al.) or *via* metabolomics (Senges et al., 2018) and of course within biotechnological studies. It was found that the lifestyle and the development stage seem to be crucial for secondary metabolism. Spore formation among *Streptomyces* is such a specialized development stage and of importance for cell regulatory processes, but also with respect to applications (Bobek et al.). Further, some regulatory elements are solely present among actinobacteria and need to be functionally tested (Koepff et al.; López-García et al.; Šetinová et al.). Growth limiting conditions (Fe-, N-, S-limitations or presence of toxic compounds/elements) are often used to overproduce target compounds and among those the secondary metabolites siderophores (Retamal-Morales et al., 2017, 2018b; Senges et al., 2018) and biosurfactants (Kügler et al., 2015; Retamal-Morales et al., 2018a) can be mentioned.

Actinobacteria also harbor extremophile branches, which become more and more attractive for biotechnological investigations (Shivlata and Satyanarayana, 2015). Examples include antimicrobial compound producers as many *Streptomyces* spp. (Radhakrishnan et al., 2007; Xue et al., 2013), siderophore producing strains as *Thermobifida fusca* (Dimise et al., 2008) and *Thermocrispum agreste* (Heine et al., 2017), and many rhizosphere specialists with various interactions toward plants, fungi and/or other bacteria (Palaniyandi et al., 2013). Besides the above described actinobacteria mainly derived from soil, also other habitats and ecological niches are explored and successfully conquered by various actinobacteria. Among those interesting resources for biotechnology are present (Shivlata and Satyanarayana, 2015).

In conclusion, it becomes obvious that the large and diverse group of actinobacteria is of interest from different perspectives such as general microbiology, ecology, phylogeny, biochemistry, and regulation, environmental concerns, pathogenicity as well as biotechnology. Still there are new members being discovered that belong to this phylum or reclassifications occur according to new findings with respect to morphology and phylogeny. The increasing amount of data from various omics fields allows us to uncover more and more properties which can be of use for various (biotechnological) purposes. We believe that the potential of actinobacteria for biotechnology was only touched lightly thus far: there is more to be uncovered!

## Author Contributions

DT, WB, and MF drafted this editorial together and approved it prior submission.

### Conflict of Interest Statement

The authors declare that the research was conducted in the absence of any commercial or financial relationships that could be construed as a potential conflict of interest.
